# Hydrophobic pore space constituted in macroporous ZIF-8 for lipase immobilization greatly improving lipase catalytic performance in biodiesel preparation

**DOI:** 10.1186/s13068-020-01724-w

**Published:** 2020-05-13

**Authors:** Yingli Hu, Lingmei Dai, Dehua Liu, Wei Du

**Affiliations:** 1 Key Laboratory for Industrial Biocatalysis, Ministry of Education, Department of Chemical Engineering, Tsinghua University, Beijing, 100084 China; 2 Tsinghua Innovation Center in Dongguan, Dongguan, 523808 Guangdong China

**Keywords:** Biodiesel, Hydrophobic macropore, Immobilization, Lipase, Metal–organic frameworks (MOFs)

## Abstract

**Background:**

During lipase-mediated biodiesel production, by-product glycerol adsorbing on immobilized lipase is a common trouble that hinders enzymatic catalytic activity in biodiesel production process. In this work, we built a hydrophobic pore space in macroporous ZIF-8 (named as M-ZIF-8) to accommodate lipase so that the generated glycerol would be hard to be adsorbed in such hydrophobic environment. The performance of the immobilized lipase in biodiesel production as well as its characteristics for glycerol adsorption were systematically studied. The PDMS (polydimethylsiloxane) CVD (chemical vapor deposition) method was utilized to get hydrophobic M-ZIF-8-PDMS with hydrophobic macropore space and then ANL (*Aspergillus niger* lipase) was immobilized on M-ZIF-8 and M-ZIF-8-PDMS by diffusion into the macropores.

**Results:**

ANL@M-ZIF-8-PDMS presented higher enzymatic activity recovery and better biodiesel production catalytic performance compared to ANL@M-ZIF-8. Further study revealed that less glycerol adsorption was observed through the hydrophobic modification, which may attribute to the improved immobilized lipase performance during biodiesel production and ANL@M-ZIF-8-PDMS remained more than 96% activity after five cycles’ reuse. Through secondary structure and kinetic parameters’ analysis, we found that ANL@M-ZIF-8-PDMS had lower extent of protein aggregation and twice catalytic efficiency (*V*_max_/*K*_m_) than ANL@M-ZIF-8.

**Conclusions:**

Hydrophobic pore space constituted in macroporous ZIF-8 for lipase immobilization greatly improved lipase catalytic performance in biodiesel preparation. The hydrophobic modification time showed negligible influence on the reusability of the immobilized lipase. This work broadened the prospect of immobilization of enzyme on MOFs with some inspiration.

## Background

In the past two decades, metal–organic frameworks (MOFs) have appeared to be a newly rising research field, due to their outstanding characteristics, like versatile structural tailorability, diversity, extremely high surface area, crystallinity and so on [1–3]. As a result, MOFs have been widely applied in various research fields [4]. And MOFs also have great potential to perform as a great research platform for enzyme immobilization based on their outstanding characteristics. Herein, enzyme immobilization on MOFs has drawn increasing research interests in recent years, and it has been demonstrated that many enzyme/MOF composites showed much better stability and catalytic performance [5–11]. Among them, MOFs were most frequently utilized for the immobilization of peroxidase and trypsin, while much less attention are paid on lipase so far [12–19].

Since lipase is a kind of important enzyme applied in various fields especially in biodiesel production (lipase catalysis method), the immobilization of lipase on MOFs is worth studying for promoting its further application. Enzyme/MOF composites are typically synthesized through three approaches, including surface immobilization, pore adsorption (diffusion into the pores of MOFs), and encapsulation of enzymes within MOFs [20–22]. Nadar et al. have activated the lipase in the presence of proline and successfully immobilized into zeolitic imidazolate framework (ZIF)-8 by biomineralization method. The prepared lipase–proline MOF exhibited 135% enhanced catalytic activity as compared to free counterpart. A highly porous lipase-loaded MOF composite was synthesized via biomimetic mineralization of ZIF-8 around lipase from Candida rugosa (CRL) with increasing enzyme loading amount [21]. The mostly applied strategy for lipase immobilization on MOFs is surface immobilization, because lipases can hardly get access to the micropores of most MOFs. Herein, the porosity of MOFs cannot be fully utilized since the internal pores are inaccessible, leading to rather poor enzyme immobilization efficiency [14, 20]. The surface immobilization strategy usually cannot bring enough stability enhancements in some respects. While other immobilization approaches like in situ encapsulation are just suitable for enzymes in catalyzing small molecule substrates, due to the pore size limitation of MOFs. Hence, constructing MOFs with larger pores (mesopore/macropore) may solve the problem encountered in lipase immobilization.

Previously, very limited works reported the lipase immobilization in mesoporous MOFs. The immobilization of *Bacillus subtilis* lipase in the mesopores of hierarchically porous MOF (with average mesopore size about 34 nm) was reported by Liu et al. [16]. And better enzymatic activity and reusability were achieved in catalyzing the esterification of small molecules like lauric acid and benzyl alcohol. Thus, further increasing the pore size range of MOFs to macropores and using it for lipase immobilization are necessary for promoting lipase practical application in catalyzing acylation reaction of long-chain fatty acid glycerides for biodiesel production. Though lipase immobilized in macroporous MOF can be enhanced in activity and stability, the common problem of glycerol accumulation in biodiesel production should still be an obstacle [23]. Considering the hydrophilic property of glycerol may result in the adsorption on the lipase, hydrophobic modification on the carrier of the immobilized lipase may lessen the adsorption of glycerol to a great extent. Despite of the fact that some researches observed the performance improvement of immobilized lipase by enhancing the hydrophobicity of supports mainly through influencing enzyme conformation [24–26], the influence on glycerol adsorption during biodiesel production has not been reported.

In this work, we tried to build a hydrophobic macropore space in macroporous ZIF-8 to accommodate lipase so that the generated glycerol would be hard to adsorb in such hydrophobic environment. Meanwhile, the hydrophobic modification might cause some enhancement of lipase by hydrophobic interaction. Normal ZIF-8 is a widely used stable MOF with only micropores of 1.1 nm and the ordered macroporous ZIF-8 (M-ZIF-8) was synthesized by the hard template method [27]. Then, hydrophobic M-ZIF-8-PDMS was synthesized by PDMS CVD method [28] to get the hydrophobic macropore space. Subsequently, the influences of hydrophobic macropore space for lipase immobilization efficiency as well as the catalytic performance in catalyzing biodiesel production were evaluated. ANL (*Aspergillus niger* lipase) was immobilized on M-ZIF-8 and M-ZIF-8-PDMS by diffusion into the macropores. The specific enzymatic activity, activity recovery, and biodiesel production catalytic performance of ANL@M-ZIF-8 and ANL@M-ZIF-8-PDMS were compared. And their difference in lipase structure and catalytic kinetic behavior caused by hydrophobic modification were also analyzed.

## Results and discussion

### Characterization of macroporous ZIF-8 (M-ZIF-8) and hydrophobic M-ZIF-8-PDMS

**Fig. 1:**
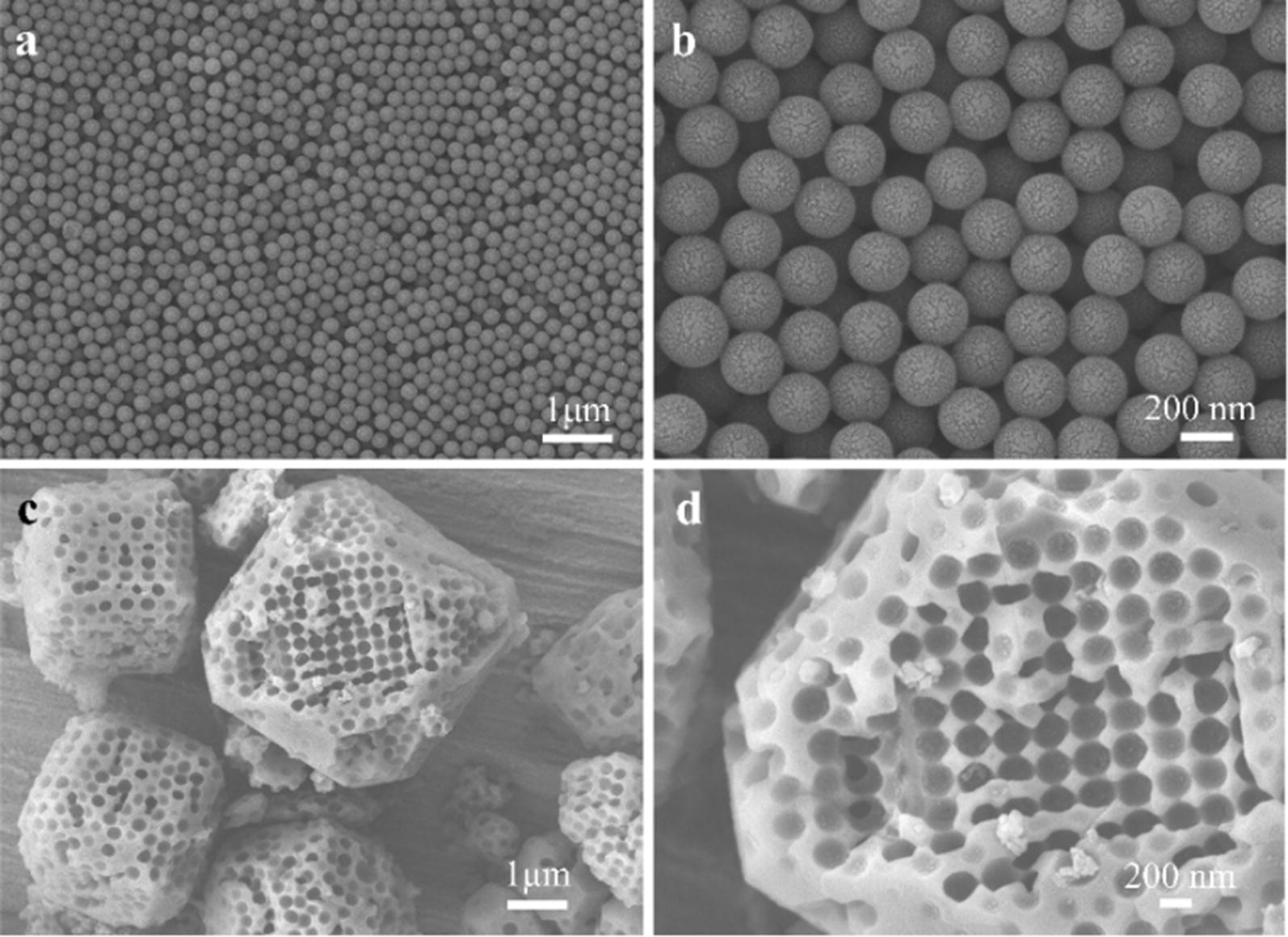
**a**, **b** SEM images of monodisperse polystyrene spheres (~ 230 nm), **c**, **d** SEM images of M-ZIF-8 (macropore diameter ~ 200 nm)

First, M-ZIF-8 was synthesized by the hard template method [27] and the assembled polystyrene (PS) nanosphere monolith was utilized as a template to get M-ZIF-8 with highly oriented and ordered macropores. Through the field emission scanning electron microscopy (SEM) images (Fig. 1a, b), the PS sphere diameter was found to be ~ 230 nm. After the etching of PS, the macropore size of ordered macro–microporous ZIF-8 was displayed as ~ 200 nm (Fig. 1c, d).

**Fig. 2:**
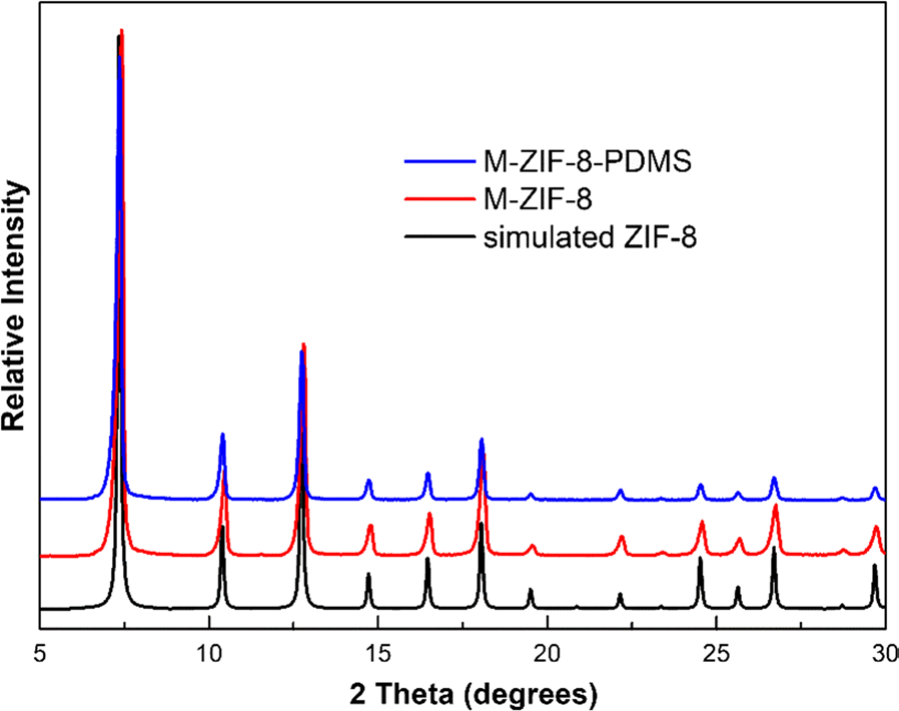
PXRD patterns of M-ZIF-8 and M-ZIF-8-PDMS with simulated ZIF-8 as comparison

Then, M-ZIF-8 was treated by PDMS CVD method and turned into hydrophobic M-ZIF-8-PDMS with a hydrophobic PDMS coating. The crystalline and micropore structure of M-ZIF-8 and M-ZIF-8-PDMS were ascertained by powder X-ray diffraction (PXRD) and N_2_ sorption isotherms’ characterization. The PXRD pattern of synthesized M-ZIF-8 and M-ZIF-8-PDMS displays sharp characteristic peaks indexed to ZIF-8, demonstrating the correct structure of M-ZIF-8 and the retention of M-ZIF-8 crystallinity after hydrophobic modification by CVD (Fig. 2).

**Fig. 3:**
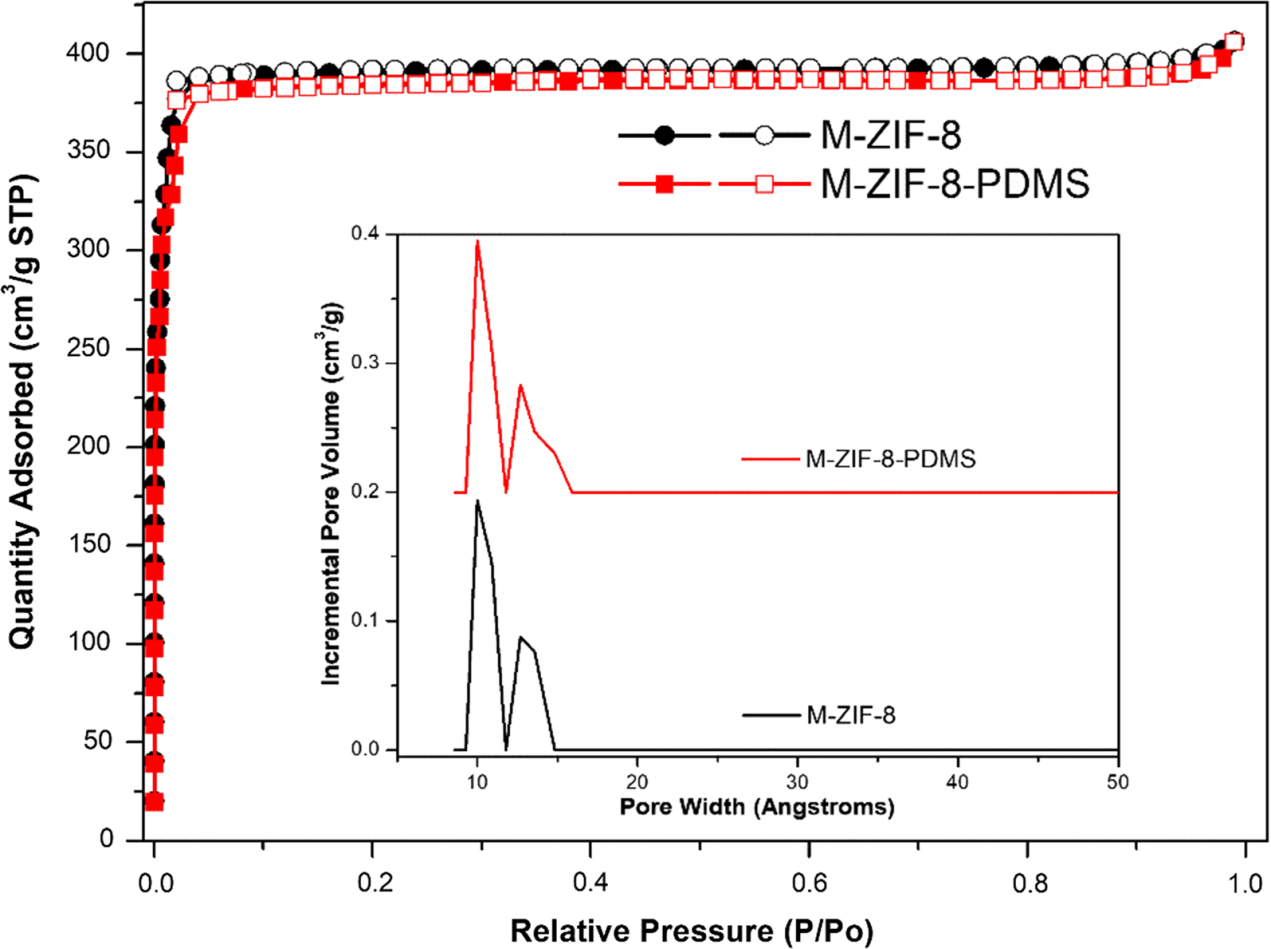
N_2_ sorption isotherms of M-ZIF-8 and M-ZIF-8-PDMS at 77 K, The inset shows the corresponding micropore size distribution from the DFT model

It was found that the N_2_ physisorption isotherms (77 K) of M-ZIF-8 and M-ZIF-8-PDMS are similar in the shape and BET surface area as an auxiliary proof of their uniformity in micropore structure (Fig. 3). Further analysis by DFT pore size distribution also supports that M-ZIF-8 and M-ZIF-8-PDMS possess similar pore size distribution in micropore scale (Fig. 3 inset).

According to the synthetic method, the macropore size of M-ZIF-8 was ~ 200 nm. While during the PDMS CVD process, PDMS decomposed into volatile silicone molecules in the form of short PDMS chains and formed a conformal layer on both M-ZIF-8 outer surface and inner surface of macropores [28]. As silicone molecules appear to be vapor, they are obviously small enough to get access into the large 200-nm macropores of M-ZIF-8. Subsequently silicone molecules crosslink, to result in the formation of hydrophobic silicone coating. To further approve the deduction, the Si element analysis of M-ZIF-8-PDMS and ZIF-8-PDMS was conducted by ICP-AES (inductively coupled plasma atomic emission spectrometry). Since M-ZIF-8 and ZIF-8 were treated in the same CVD conditions, M-ZIF-8-PDMS should possess more Si element content (represent PDMS coating content) compared to ZIF-8-PDMS, due to its extra inner surface of macropores. And the result was just as expected, M-ZIF-8-PDMS presented higher Si content than ZIF-8-PDMS (3.8 vs 2.9 mg/g).

### Comparative study of immobilizing lipase on M-ZIF-8 and hydrophobic M-ZIF-8

**Fig. 4:**
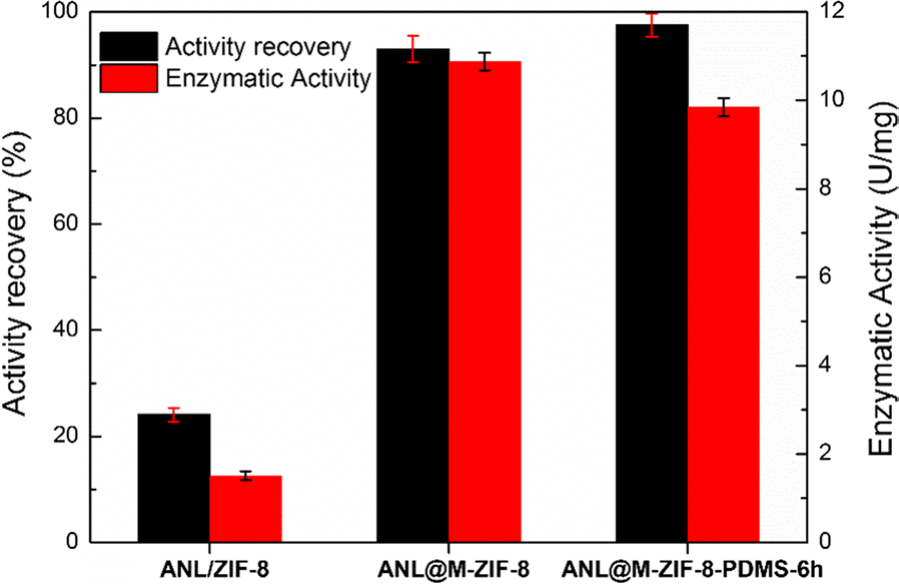
The activity recovery (black) and specific enzymatic activity (red) of lipase

After the structure characterization, M-ZIF-8 and M-ZIF-8-PDMS-6 h were applied to immobilize *Aspergillus niger* lipase (ANL) by physical adsorption, and ANL directly diffused into their macropores. It was found that both ANL@M-ZIF-8 and ANL@M-ZIF-8-PDMS-6 h presented significant improvements of specific enzymatic activity and activity recovery compared to ANL/ZIF-8 (Fig. 4) due to the difference of pore adsorption (ANL on M-ZIF-8 and M-ZIF-8-PDMS-6 h) and surface immobilization (ANL on conventional ZIF-8). Further comparing ANL@M-ZIF-8 and ANL@M-ZIF-8-PDMS-6 h, the latter showed higher enzymatic activity recovery due to the hydrophobic interaction. And the apparent specific enzymatic activity of ANL@M-ZIF-8-PDMS-6 h was a little lower than ANL@M-ZIF-8 as a result of lower loading efficiency (51.2% vs 59.7%). This phenomenon could be explained by the fact that the ANL in aqueous solution was harder to diffuse into hydrophobic macropores of M-ZIF-8-PDMS-6 h than macropores of M-ZIF-8. This result indicated that the hydrophobicity of M-ZIF-8 has an influence on the immobilization of ANL on M-ZIF-8 through macropore adsorption.

### Comparative study on ANL@M-ZIF-8 and ANL@M-ZIF-8-PDMS in biodiesel production

**Fig. 5:**
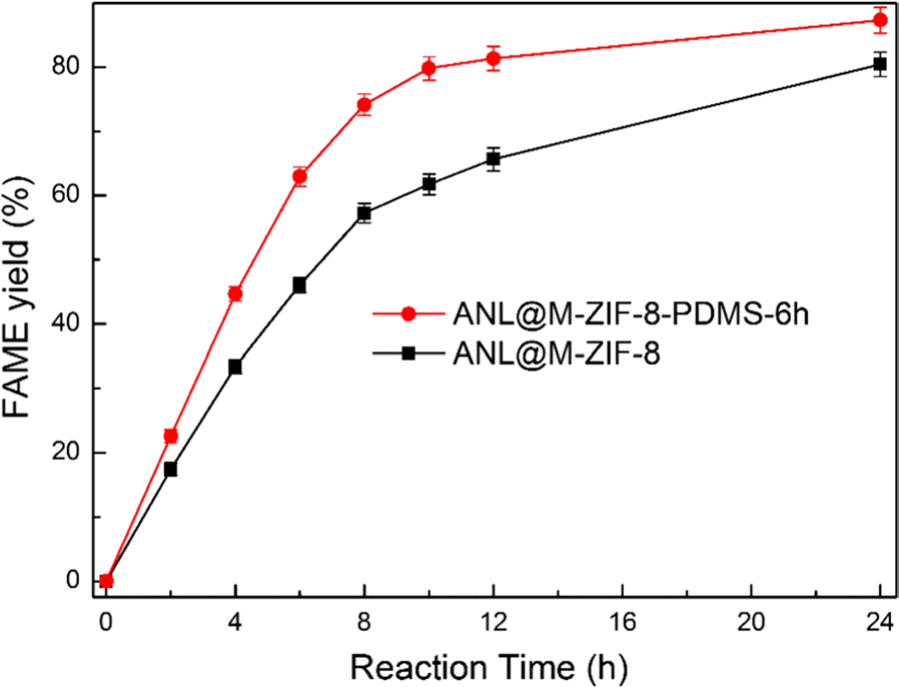
Catalytic performances of ANL@M-ZIF-8-PDMS-6 h and ANL@M-ZIF-8 in biodiesel production

To further study the effect of hydrophobic modification to M-ZIF-8, the catalytic performances of ANL@M-ZIF-8 and ANL@M-ZIF-8-PDMS in biodiesel production were investigated comparatively. It was found that ANL@M-ZIF-8-PDMS presented a little higher reaction rate than ANL@M-ZIF-8 since the primary stage and finally reached 88% of FAME yield at 24 h, while ANL@M-ZIF-8 reached 80% (Fig. 5). The improvement could be attributed to the better mass transfer of oil molecules in the hydrophobic macropores of ANL@M-ZIF-8-PDMS-6 h and less-adsorbed by-product glycerol in the reaction process compared to ANL@M-ZIF-8. To prove the adsorbed glycerol assumption, the amount of adsorbed glycerol after reaction was measured (total generated glycerol minus glycerol in solution). The result showed ANL@M-ZIF-8-PDMS-6 h adsorbed less glycerol (36.2 mg, 38.8% adsorbed proportion) than ANL@M-ZIF-8 (44.1 mg, 50.7% adsorbed proportion) while it produced more glycerol in total. This verified that hydrophobic modification could reduce the affinity of immobilized lipase with glycerol. To further prove the influence of adsorbed glycerol on reaction process, a series of ANL@M-ZIF-8-PDMS-6 h catalyzed biodiesel production reactions were led with addition of gradient-increased glycerol before reaction. As a result, the final FAME yield gradually declined with the increase of pre-added glycerol. In summary, the adsorbed glycerol is an important inhibition factor of ANL@M-ZIF-8 and ANL@M-ZIF-8-PDMS catalyzed biodiesel production reaction. The by-product glycerol influences the immobilized lipase reusability and catalytic performance mainly through influencing the mass transfer of reactants and products.

**Fig. 6:**
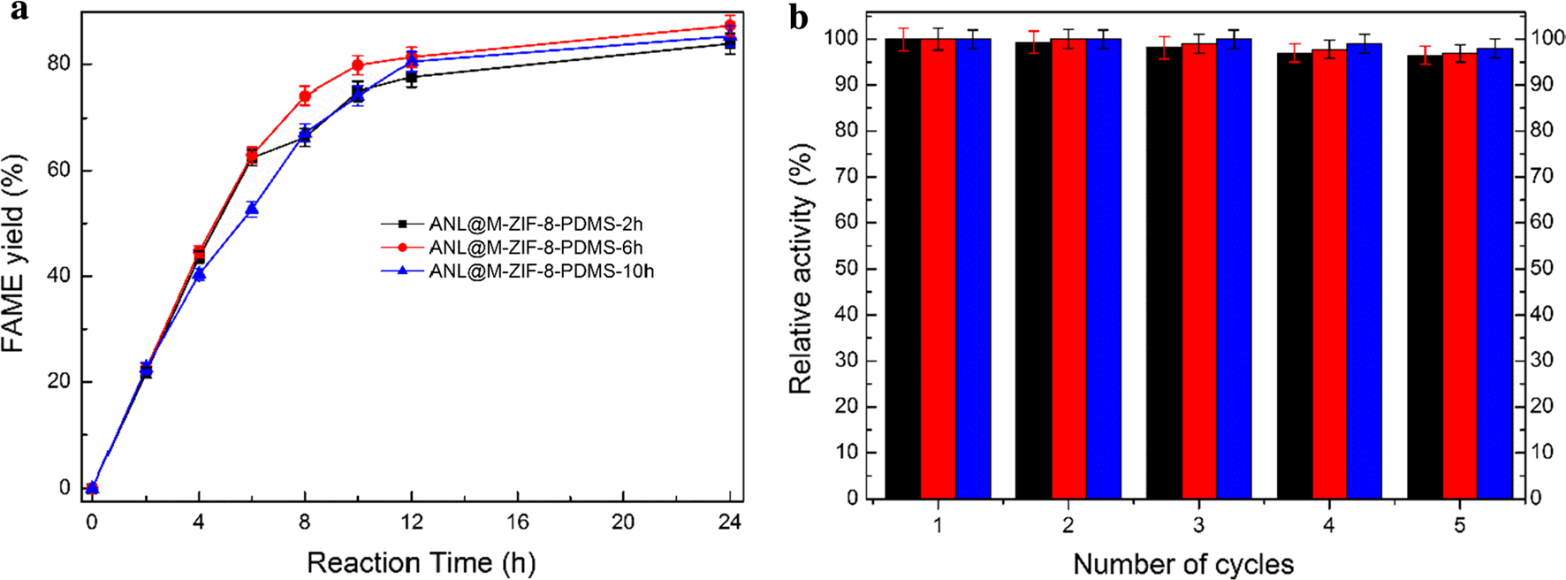
Catalytic performances (**a**) and reusability (**b**) of ANL@M-ZIF-8-PDMS with different hydrophobicity (-2 h, -6 h, -10 h) in biodiesel production

Since ANL on hydrophobic M-ZIF-8-PDMS-6 h could efficiently reduce the amount of adsorbed glycerol, M-ZIF-8 supports with various hydrophobicities were also utilized to immobilize ANL. And their catalytic performances were compared. As shown in Fig. 6, ANL@M-ZIF-8-PDMS-2 h, ANL@M-ZIF-8-PDMS-6 h and ANL@M-ZIF-8-PDMS-10 h presented similar FAME yield, and excellent reusability (more than 96% activity remained) after five cycles. This might suggest that hydrophobic modification of M-ZIF-8 could improve catalytic performance of ANL@M-ZIF-8 while the extent of hydrophobic modification was of little influence.

### Enzyme structure and kinetic behavior analysis of ANL@M-ZIF-8 and ANL@M-ZIF-8-PDMS

**Fig. 7:**
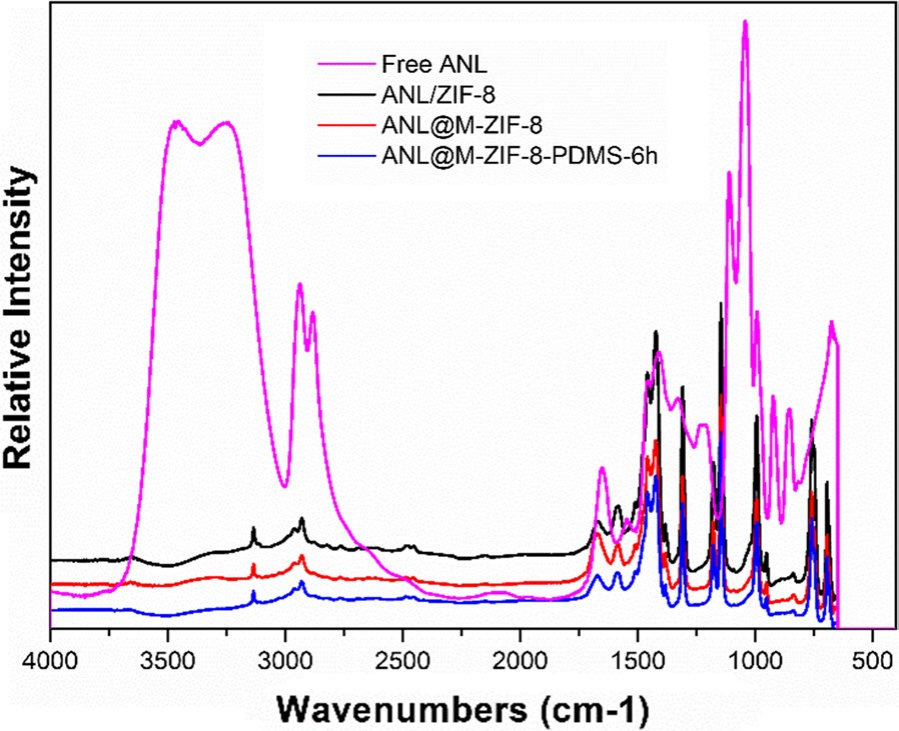
The FTIR spectra of free ANL, ANL/ZIF-8, ANL@M-ZIF-8 and ANL@M-ZIF-8-PDMS-6 h

**Fig. 8:**
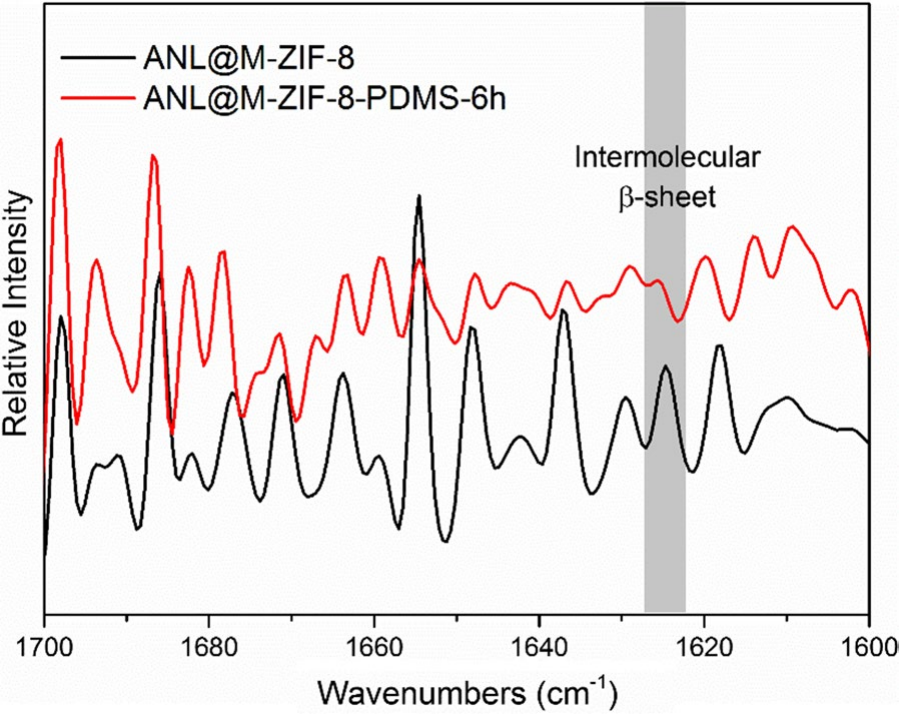
The second derivatives of the FTIR spectra of ANL@M-ZIF-8-PDMS-6 h and ANL@M-ZIF-8 in amide I (1600–1700 cm^−1^) bands

To verify how hydrophobicity of M-ZIF-8 influenced the structure of immobilized ANL, ATR/FTIR (Attenuated total reflectance/Fourier transform infrared) spectroscopy was used to quantify the secondary structure components of ANL adsorbed on M-ZIF-8 and M-ZIF-8-PDMS-6 h. And their FTIR spectra are shown in Fig. 7 along with that of free ANL and ANL/ZIF-8. As is well known, the protein structure reference spectra are generated in the amide I (1600–1700 cm^−1^) and amide III (1200–1300 cm^−1^) bands. The amide I band estimates are usually better than the amide III band estimates. Therefore, the amide I band was used herein. A second derivative analysis to locate peaks due to secondary structural components was adopted [24, 29]. After second derivative analysis of amide I (1600–1700 cm^−1^) bands, the second derivatives of the spectra are shown in Fig. 8. The mean peak positions were α-helix (1656 cm^−1^), intramolecular β-sheet (1693 and 1633 cm^−1^), unordered and ordered helix (1672, 1648 and 1620 cm^−1^) and turn structures (1720, 1668, 1630 and 1617 cm^−1^). Notably, the peaks at 1627 cm^−1^ and 1622 cm^−1^ indicated the formation of intermolecular contacts [22]. According to Fig. 8, the intermolecular β-sheet peak decreased when the surface became more hydrophobic. In other words, the extent of protein aggregation in ANL@M-ZIF-8-PDMS-6 h decreased compared to ANL@M-ZIF-8.

The kinetic behavior of ANL@M-ZIF-8-PDMS-6 h and ANL@M-ZIF-8 in biodiesel production was compared (Table 1). Kinetic parameters were determined by measuring initial reaction rates for each form with varying concentration of methanol. It was found that the ANL-catalyzed methanolysis of soybean oil followed the Michaelis–Menten model. The apparent *V*_max_ of the ANL@M-ZIF-8-PDMS-6 h was higher than that of ANL@M-ZIF-8 (0.526 vs 0.393 mM min^−1^). And the apparent kinetic parameter *K*_m_ of ANL@M-ZIF-8-PDMS-6 h was lower than that of ANL@M-ZIF-8 (1.32 × 10^3^ vs 1.89 × 10^3^ mM), demonstrating an increase in apparent enzyme–substrate affinity after hydrophobic modification of M-ZIF-8.49. In addition, the catalytic efficiency *V*_max_/*K*_m_ value of ANL@M-ZIF-8-PDMS-6 h was higher than that of ANL@M-ZIF-8 (3.97 × 10^−4^ vs 2.07 × 10^−4^ min^−1^). The possible explanation for this observation was that the three-dimensional structure of ANL underwent a conformational change on hydrophobic M-ZIF-8-PDMS-6 h support compared to M-ZIF-8, making it easier for the substrate to access the lipase active site. Besides, the hydrophobic modification of M-ZIF-8 also led to better mass transfer in oil phase, which benefited the increase of *V*_max_ and catalytic efficiency *V*_max_/*K*_m_.

**Table 1:** Kinetic parameters of ANL@M-ZIF-8 and ANL@M-ZIF-8-PDMS-6 h

Lipase	*V*_max_ (mM min^−1^)	*K*_m_ (mM)	*V*_max_/*K*_m_ (min^−1^)
ANL@M-ZIF-8-PDMS-6 h	0.526	1.32 × 10^3^	3.97 × 10^−4^
ANL@M-ZIF-8	0.393	1.89 × 10^3^	2.07 × 10^−4^

## Conclusions

This work systematically studied the influence of hydrophobic modification on macroporous MOF immobilizing lipase, and proposed an effective way to solve the by-product glycerol adsorbing problem in biodiesel production process. By building hydrophobic macropore space, we found that the catalytic performance of ANL@M-ZIF-8-PDMS was remarkably improved compared to ANL@M-ZIF-8 during biodiesel production. The improved catalytic activity as well as the reusability of the immobilized lipase with hydrophobic macropore space were mainly due to the hydrophobic interaction and the decreased glycerol affinity. And ANL@M-ZIF-8-PDMS remained more than 96% activity after five cycles’ reuse. Through secondary structure and kinetic parameters analysis, we found that ANL@M-ZIF-8-PDMS had lower extent of protein aggregation and twice catalytic efficiency (*V*_max_/*K*_m_) than ANL@M-ZIF-8. This work broadened the prospect of immobilization of enzyme on MOFs with some inspiration.

## Materials and methods

### Materials

Lipase from the genetically modified *Aspergillus niger* was donated by Novozymes (Copenhagen, Denmark). Tributyrin was purchased from Tokyo Chemical Industry, Japan. The bicinchoninic acid (BCA) protein assay was purchased from Beijing leagene biotech.co. Ltd, China. Heptadecanoic acid methyl ester was purchased from Sigma-Aldrich (St. Louis, MO), while ethanol and methanol were purchased from Beijing Chemical works Co. Ltd, China. Soybean oil was purchased from the local market. All the other chemicals were purchased commercially with analytical grade.

### Characterization methods

Powder X-ray diffraction (PXRD) patterns were tested by a Bruker D8 Advance X-Ray diffractometer with a Cu Kα anode (λ = 0.15406 nm) at 40 kV and 40 mA. N_2_ adsorption–desorption isotherms were measured at 77 K on a Micromeritics ASAP 2020 analyzer. The samples were degassed at 80 °C for 12 h before the measurements. Specific surface areas were calculated by Brunauer–Emmett–Teller (BET) method in the relative pressure range P/P_0_ = 0.05–0.30. The FTIR (Fourier transform infrared) spectra were collected in the 1000–4000 cm^−1^ range by a Nicolet 6700FTIR. The reaction process was analyzed through Agilent 7890A gas chromatograph (GC), which was equipped with CP-FFAP capillary column (0.32 mm × 0.30 μm × 25 m). The initial column temperature was 180 °C and maintained for 0.5 min, then the column was heated to 250 °C at the rate of 10 °C/min and held for 6 min. Detector and injector were set at 250 °C and 245 °C, respectively.

### Experimental methods

#### Preparation of 3D ordered polystyrene (PS) template

The synthesis was according to the reported procedure with modification [27]. 65 mL washed styrene (removal of stabilizer) and 500 mL aqueous solution of PVP (k-30, 2.50 g) were added to 1-L triple-neck round-bottomed flask. After bubbling with nitrogen for 15 min, the mixture was heated at 75 °C for 30 min under mechanical stirring (450 rpm). Subsequently, to initiate the polymerization reaction, 50 mL of K_2_S_2_O_8_ (1.00 g) aqueous solution was added quickly into the flask, and the reaction lasted for 24 h at 75 °C, 450 rpm stirring. After reaction, the mixture was cooled down and poured onto a filter funnel with two conventional filter papers under vacuum. After filtering for ~ 24 h, the formed filter cakes were washed by deionized water and ethanol, and then dried in 60 °C oven overnight.

### Synthesis of single-crystalline ordered macroporous ZIF-8 (M-ZIF-8)

Based on reported method with modification [27], sufficient precursor methanol solution was prepared according to the ratio of Zn(NO_3_)_2_·6H_2_O (8.15 g), 2-methylimidazole (6.75 g) and methanol (45 mL). Then, the PS template (filter cake) was soaked into the above solution for 1 h and further degassed in vacuum for 10 min. After the soaked filter cake was dried at 50 °C for 12 h, it was then soaked in CH_3_OH/NH_3_·H_2_O (1:1 v/v) mixed solution at room temperature (RT). This mixture was degassed in vacuum for 10 min and then reacted at RT and atmospheric pressure for 24 h. The filter cake gradually broke into small pieces due to the growing stress of ZIF-8 and they were filtrated and dried in air after reaction. Then, PS templates confined in M-ZIF-8 were removed by soaking in tetrahydrofuran for 24 h. To ensure the thorough etching of PS, this process was repeated > 5 times. Finally, the obtained white powder was vacuum dried at 100 °C overnight.

### Synthesis of M-ZIF-8-PDMS

The PDMS coated M-ZIF-8 (named as M-ZIF-8-PDMS) was prepared by a simple CVD (Chemical Vapor Deposition) method based on reported literature [30] with modification. M-ZIF-8 powder was flat in glass dish as thin layer, and then placed in a vacuum glass container with some newly prepared PDMS pieces in the bottle. The glass container was heated at 200 °C for 6 h in an oven and then cooled down naturally to yield M-ZIF-8-PDMS. Samples with different hydrophobicity can be obtained by changing CVD treating time (2 h, 6 h, 10 h), which were named as M-ZIF-8-PDMS-2 h, M-ZIF-8-PDMS-6 h, and M-ZIF-8-PDMS-10 h.

### Immobilization of *Aspergillus niger* lipase (ANL)

First, 60 mg supports (M-ZIF-8 or M-ZIF-8-PDMS) and 800 μL deionized water were added into 2-mL plastic centrifuge tube, and the mixture was ultrasonicated to uniform dispersion. Then 200 μL free lipase ANL was added into the mixture. The mixture was placed in a thermostatic shaker at 45 °C, 200 rpm for > 4 h. After that, the immobilized lipase was collected by centrifugation or vacuum filtration (by filter membrane) and washed with water once. Finally, the sample was dried through lyophilization. The loading amount of lipase was calculated by detecting the protein concentration of supernatant and free lipase through BCA (bicinchoninic acid) method (using BCA Protein Assay Kit).

$${\text{Loading amount }}L = \frac{{\left( {C_{0} - C_{1} } \right) \times V}}{{m_{\text{s}} }}$$

where *C*_0_ and *C*_1_ represent the lipase protein concentration of supernatant before and after immobilization, respectively, *V* is the volume of lipase solution, and *m*_s_ the weight of support.

### Standard determination of enzyme activity

The specific activity of the free and immobilized lipase was measured by butyrin hydrolysis method according to previous reports [31]. All data are from triplicated experiments.

### Lipase-catalyzed methanolysis of soybean oil

The reaction was performed at the conditions as follows: 10 g soybean oil, 1 g water, an appropriate amount of immobilized enzyme (~ 0.2–0.5 g) with equal enzymatic activity of 120 U per gram soybean oil, were added in a 50-mL Erlenmeyer flask which was placed in thermostatic shaker at 45 °C, 200 rpm. Then, methanol was added through stepwise addition (total mole ratio of methanol/oil = 4/1) of four steps (460 μL × 4) at 0 h, 2 h, 4 h, and 6 h. At different intervals, 50-μL sample was taken for the GC (gas chromatography) analysis of FAMEs (fatty acid methyl esters) content. The sample was first treated by speed vacuum concentrator at 85 °C, 2000 rpm, − 0.1 MPa. Then ~ 10 μL weighed sample and 600 μL heptadecanoic acid methyl ester ethanol solution (internal standard, 0.8 g/L) were mixed and taken for GC) analysis. The FAME yield was calculated by the following formula:

$${\text{FAME yield }}\left( {\text{\% }} \right) = \frac{{m_{\text{i}} \times A_{\text{s}} }}{{A_{\text{i}} \times m_{\text{s}} }} \times 100$$

where *m*_i_ and *m*_s_ represent the mass of internal standard and sample, respectively, *A*_i_ and *A*_s_ represent the GC peak area of internal standard and FAMEs, respectively. All data are from triplicated experiments.

GC analysis conditions: FID (Agilent 7890A) and a column (CB-FFAP (0.32 mm × 2 m; Chromapack) DB-1 (0.25 mm × 15 m; J&W Scientific, Folsom, CA) were used to carry out the analysis. The initial column temperature was set at 180 °C and held for 0.5 min, then heated to 250 °C at the rate of 10 °C/min and held for 6 min. The temperature of detector and injector was set at 250 °C and 245 °C, respectively.

### Reusability test of ANL@M-ZIF-8 in methanolysis of soybean oil

The reusability reaction was the same as above and 50-μL sample was taken from the system at 12 h for GC analysis. Then, after each batch reaction finished, the immobilized lipase was collected by centrifuging (10,000 rpm) and washed thoroughly by t-butanol for next batch catalyzing. All data are from triplicated experiments.

### Determination of kinetic parameters in methanolysis of soybean oil

Kinetic parameters were determined with varied concentrations of methanol from 0.05 to 0.20 M in soybean oil at 45 °C. In each case, 10 mg of immobilized lipase was used, and the reactions were ended at 10 min. The apparent *K*_m_ and *V*_max_ (kinetic parameters) values were calculated from Hanes–Woolf plots.

## Data Availability

The data supporting the results of the article are included in this manuscript.

## Abbreviations

PDMS: Polydimethylsiloxane
CVD: Chemical vapor deposition
ANL: *Aspergillus niger* lipase
MOFs: Metal–organic frameworks
BCA: Bicinchoninic acid
PXRD: Powder X-ray diffraction
BET: Brunauer–Emmett–Teller
FTIR: Fourier transform infrared
PS: Poly styrene
PVP: Polyvinyl pyrrolidone
RT: Room temperature
M-ZIF-8-PDMS: PDMS coated M-ZIF-8
CVD: Chemical vapor deposition
GC: Gas chromatography
FAMEs: Fatty acid methyl esters
SEM: Scanning electron microscopy
PXRD: Powder X-ray diffraction
ICP-AES: Inductively coupled plasma atomic emission spectrometry
ATR/FTIR: Attenuated total reflectance/Fourier transform infrared
ZIF: Zeolitic imidazolate framework
